# Crystal structure of 1-butyl-2,3-di­methyl­imidazolium dicarba-7,8-*nido*-undeca­borate

**DOI:** 10.1107/S2056989015002765

**Published:** 2015-02-13

**Authors:** M. J. Klemes, L. Soderstrom, J. L. Hunting, A. S. Larsen

**Affiliations:** a Department of Chemistry, Ithaca College, 953 Danby Road, Ithaca, NY 14850, USA

**Keywords:** crystal structure, carborane cage anion, imidazolium cation, bridging B—H—B bond

## Abstract

In the title mol­ecular salt, C_9_H_17_N_2_^+^·C_2_H_12_B_9_^−^, the carborane cage has a bridging B—H—B bond on the open B_3_C_2_ face. The butyl side chain of the cation adopts an extended conformation [C—C—C—C = 179.6 (1)°]. In the crystal, the imidazolium ring is almost coplanar with the open face of the carborane anion. The cations stack in the [010] direction and the dihedral angle between the imidazolium rings of adjacent cations is 68.45 (6)°. The butyl chains extend into the space between carborane anions.

## Related literature   

For structural and thermodynamic properties of the title compound and similar boron cluster anion low-melting ionic compounds, see: Larsen *et al.* (2000 ▸); Dymon *et al.* (2008 ▸); Suarez *et al.* (2011 ▸). A similar bridging hydrogen atom was reported by Jones *et al.* 1997 ▸ in an analogous crystal structure.
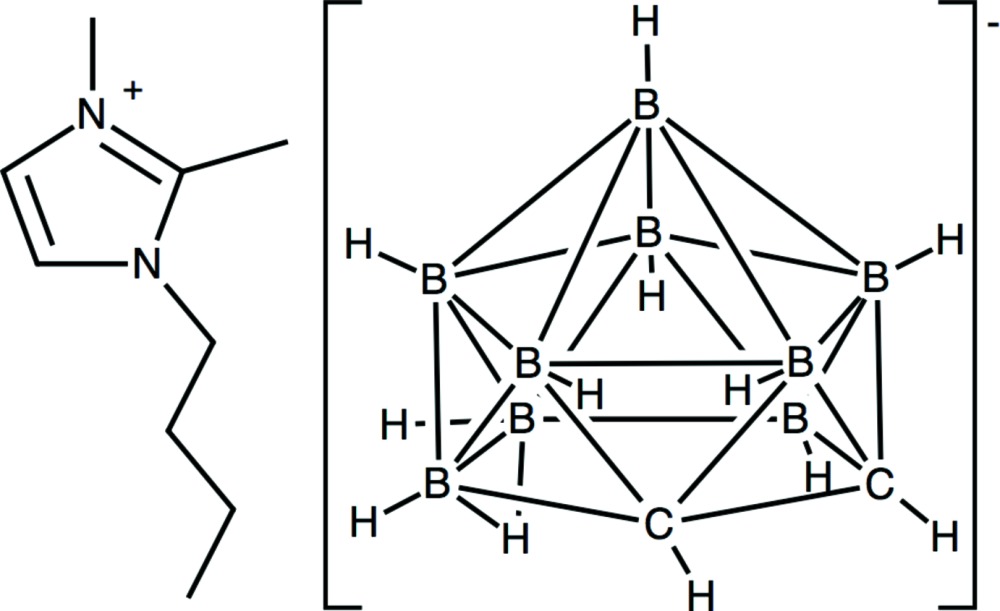


## Experimental   

### Crystal data  

C_9_H_17_N_2_^+^·C_2_H_12_B_9_^−^
*M* *_r_* = 286.78
Monoclinic, 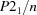

*a* = 9.5242 (2) Å
*b* = 11.5173 (2) Å
*c* = 16.3357 (3) Å
β = 104.821 (1)°
*V* = 1732.30 (6) Å^3^
*Z* = 4
Mo *K*α radiation
μ = 0.06 mm^−1^
*T* = 100 K
0.42 × 0.32 × 0.26 mm

### Data collection  

Bruker SMART CCD 1K area-detector diffractometer
Absorption correction: multi-scan (*SADABS*; Bruker, 2012 ▸) *T*_min_ = 0.701, *T*_max_ = 0.746
27028 measured reflections
3964 independent reflections
3582 reflections with *I* > 2σ(*I*)
*R*_int_ = 0.021

### Refinement  

*R*[*F* ^2^ > 2σ(*F* ^2^)] = 0.043
*wR*(*F* ^2^) = 0.120
*S* = 1.05
3964 reflections
225 parameters
H atoms treated by a mixture of independent and constrained refinement
Δρ_max_ = 0.40 e Å^−3^
Δρ_min_ = −0.28 e Å^−3^

### 

Data collection: *APEX2* (Bruker, 2012 ▸); cell refinement: *SAINT* (Bruker, 2012 ▸); data reduction: *SAINT*; program(s) used to solve structure: *SHELXS97* (Sheldrick, 2008 ▸); program(s) used to refine structure: *SHELXL2013* (Sheldrick, 2015 ▸); molecular graphics: *OLEX2* (Dolomanov *et al.*, 2009 ▸); software used to prepare material for publication: *OLEX2*.

## Supplementary Material

Crystal structure: contains datablock(s) I. DOI: 10.1107/S2056989015002765/hb7327sup1.cif

Structure factors: contains datablock(s) I. DOI: 10.1107/S2056989015002765/hb7327Isup2.hkl

**Fig. 1.**
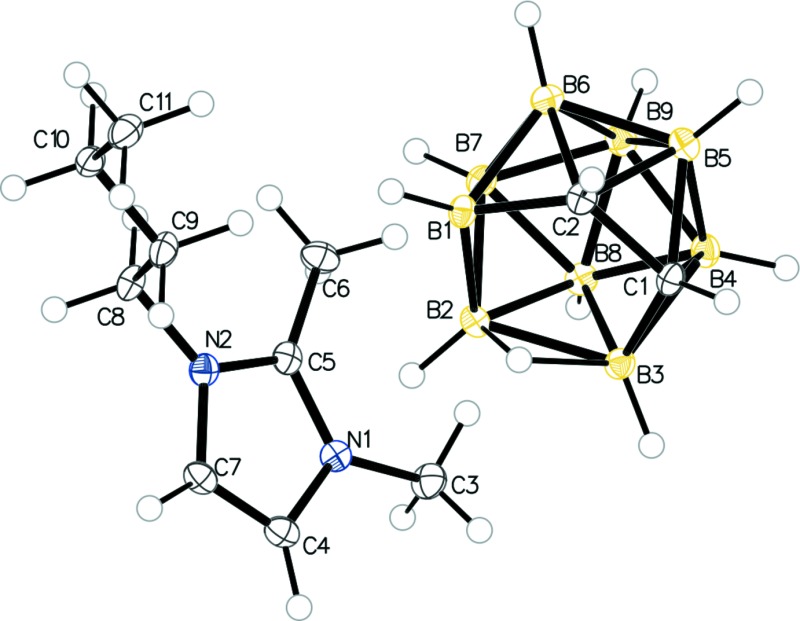
Crystal structure of the title compound with displacement ellipsoids at the 50% probability level.

. DOI: 10.1107/S2056989015002765/hb7327fig1.tif
Crystal structure of the title compound with displacement ellipsoids at the 50% probability level.

**Fig. 2.**
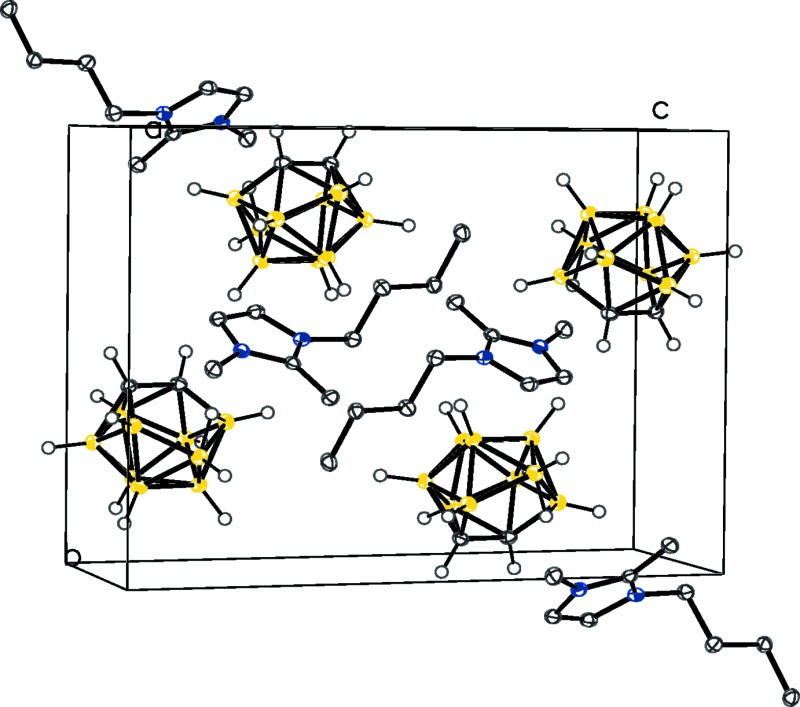
Packing diagram of the title compound, showing coplanar alignment of the imidazolium rings and parallel butyl chains. The angle between two orientations of coplanar imidazolium rings is 68.45°.

. DOI: 10.1107/S2056989015002765/hb7327fig2.tif
Packing diagram of the title compound, showing coplanar alignment of the imidazolium rings and parallel butyl chains. The angle between two orientations of coplanar imidazolium rings is 68.45°.

**Fig. 3.**
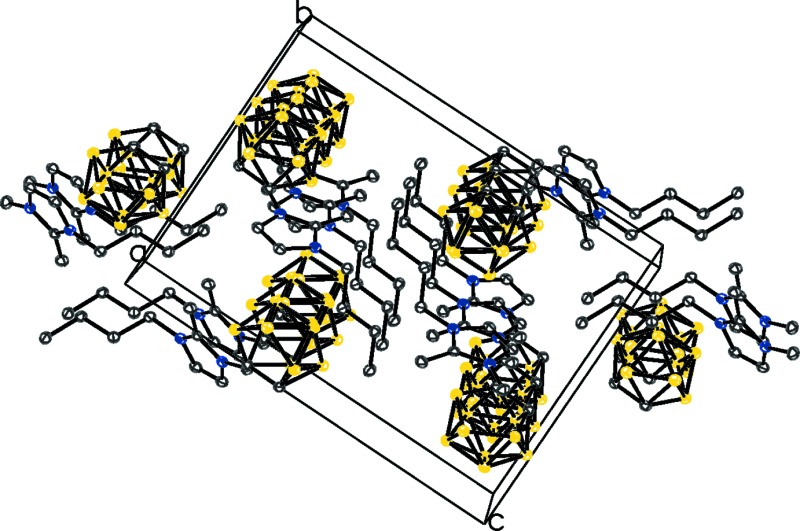
Packing diagram of the title compound showing the imidazolium rings nearly coplanar with the open B3C2 face. For clarity, H-atoms are removed.

. DOI: 10.1107/S2056989015002765/hb7327fig3.tif
Packing diagram of the title compound showing the imidazolium rings nearly coplanar with the open B3C2 face. For clarity, H-atoms are removed.

CCDC reference: 1048494

Additional supporting information:  crystallographic information; 3D view; checkCIF report

## supplementary crystallographic information

### S1. Comment

The title compound was synthesized as part of a study of low melting ionic compounds with carborane cage anions. (Larsen *et al.* 2000, Dymon *et al.* 2008, Suarez *et al.* 2011). The formula unit consists of a carborane anion and an alkylated imidazolium cation. A similar bridging hydrogen atom was also seen, for example, by Jones *et al.* 1997 in an analogous crystal structure. In the crystal packing the imidazolium rings are almost coplanar with open face of the carborane anions. The angle between two orientations of coplanar imidazolium rings is 68.45°. The butyl chains form interlinking pattern extending into the space between carborane anions. The carborane anion possesses a bridging hydrogen at the open face of the cage (shown on Figure 1).

### S2. Experimental

Caesium dicarba-7,8-*nido*-undecaborate was synthesized according to published procedure (Dymon *et al.* 2008). Caesium dicarba-7,8-*nido*-undecaborate (0.300 g, 1.14 mmol) was dissolved in acetone (2 ml). This solution was added to 1-butyl-2,3-dimethylimidazolium chloride (0.215 g, 1.14 mmol) dissolved in dichloromethane (30 ml). The turbid solution was stirred at room temperature for 30 minutes. The mixture was filtered through a plug of celite to remove the caesium chloride precipitate. The volatiles were removed *in vacuo* and the residue was dissolved in dichloromethane (30 ml) and filtered *via* celite again. The dichloromethane was removed *in vacuo*. The solid residue was dissolved in a small amount of absolute ethanol and crystals were grown by slow vapor diffusion of hexane into the absolute ethanol solution at 233 K.

### S3. Refinement

Crystal data, data collection and structure refinement details are summarized in Table 1. The methyl and aromatic H atoms were constrained to an ideal geometry; the methyl H atoms were allowed to rotate freely about the C—C bonds. The H atoms attached to B atoms were placed in calculated positions.

### Crystal data

**Table:**

C_9_H_17_N_2_^+^·C_2_H_12_B_9_^−^	*F*(000) = 616
*M**_r_* = 286.78	*D*_x_ = 1.099 Mg m^−^^3^
Monoclinic, *P*2_1_/*n*	Mo *K*α radiation, λ = 0.71073 Å
*a* = 9.5242 (2) Å	Cell parameters from 3582 reflections
*b* = 11.5173 (2) Å	θ = 2.2–27.5°
*c* = 16.3357 (3) Å	µ = 0.06 mm^−^^1^
β = 104.821 (1)°	*T* = 100 K
*V* = 1732.30 (6) Å^3^	Prism, clear light colourless
*Z* = 4	0.42 × 0.32 × 0.26 mm

### Data collection

**Table:**

Bruker SMART CCD 1K area-detector diffractometer	3964 independent reflections
Radiation source: sealed X-ray tube	3582 reflections with *I* > 2σ(*I*)
Graphite monochromator	*R*_int_ = 0.021
Detector resolution: 7.9 pixels mm^-1^	θ_max_ = 27.5°, θ_min_ = 2.2°
ω scans	*h* = −12→12
Absorption correction: multi-scan (*SADABS*; Bruker, 2012)	*k* = −14→14
*T*_min_ = 0.701, *T*_max_ = 0.746	*l* = −21→20
27028 measured reflections	

### Refinement

**Table:**

Refinement on *F*^2^	37 constraints
Least-squares matrix: full	Primary atom site location: structure-invariant direct methods
*R*[*F*^2^ > 2σ(*F*^2^)] = 0.043	H atoms treated by a mixture of independent and constrained refinement
*wR*(*F*^2^) = 0.120	*w* = 1/[σ^2^(*F*_o_^2^) + (0.0633*P*)^2^ + 0.7865*P*] where *P* = (*F*_o_^2^ + 2*F*_c_^2^)/3
*S* = 1.05	(Δ/σ)_max_ = 0.004
3964 reflections	Δρ_max_ = 0.40 e Å^−^^3^
225 parameters	Δρ_min_ = −0.28 e Å^−^^3^
0 restraints	

### Special details

**Table:**

Geometry. All e.s.d.'s (except the e.s.d. in the dihedral angle between two l.s. planes) are estimated using the full covariance matrix. The cell e.s.d.'s are taken into account individually in the estimation of e.s.d.'s in distances, angles and torsion angles; correlations between e.s.d.'s in cell parameters are only used when they are defined by crystal symmetry. An approximate (isotropic) treatment of cell e.s.d.'s is used for estimating e.s.d.'s involving l.s. planes.

### Fractional atomic coordinates and isotropic or equivalent isotropic displacement parameters (Å^2^)

**Table:**

	*x*	*y*	*z*	*U*_iso_*/*U*_eq_	
N1	0.76133 (10)	0.49244 (8)	0.20979 (6)	0.0148 (2)	
N2	0.67739 (10)	0.47136 (8)	0.31974 (6)	0.0149 (2)	
C3	0.85032 (13)	0.52932 (10)	0.15375 (7)	0.0201 (2)	
H3a	0.8045 (5)	0.5960 (5)	0.1201 (4)	0.0301 (4)*	
H3b	0.9471 (3)	0.5514 (8)	0.18769 (8)	0.0301 (4)*	
H3c	0.8590 (8)	0.4653 (3)	0.1159 (4)	0.0301 (4)*	
C6	0.90467 (12)	0.59313 (10)	0.34029 (7)	0.0195 (2)	
H6a	0.99760 (12)	0.5562 (4)	0.3408 (5)	0.0292 (4)*	
H6b	0.9000 (6)	0.6697 (3)	0.3136 (3)	0.0292 (4)*	
H6c	0.8963 (6)	0.6019 (7)	0.39853 (18)	0.0292 (4)*	
C5	0.78385 (12)	0.51986 (9)	0.29174 (7)	0.0145 (2)	
C4	0.63722 (12)	0.42556 (9)	0.18504 (7)	0.0169 (2)	
H4	0.59636 (12)	0.39499 (9)	0.13008 (7)	0.0203 (3)*	
C7	0.58539 (12)	0.41206 (9)	0.25370 (7)	0.0167 (2)	
H7	0.50115 (12)	0.36962 (9)	0.25636 (7)	0.0200 (3)*	
C8	0.66132 (12)	0.47245 (10)	0.40748 (7)	0.0165 (2)	
H8a	0.72201 (12)	0.53512 (10)	0.43998 (7)	0.0198 (3)*	
H8b	0.55882 (12)	0.48834 (10)	0.40668 (7)	0.0198 (3)*	
C9	0.70717 (12)	0.35634 (10)	0.45034 (7)	0.0178 (2)	
H9a	0.64746 (12)	0.29405 (10)	0.41672 (7)	0.0214 (3)*	
H9b	0.80982 (12)	0.34134 (10)	0.45104 (7)	0.0214 (3)*	
C10	0.69123 (12)	0.35140 (10)	0.54080 (7)	0.0164 (2)	
H10a	0.75173 (12)	0.41286 (10)	0.57497 (7)	0.0197 (3)*	
H10b	0.58876 (12)	0.36634 (10)	0.54054 (7)	0.0197 (3)*	
C11	0.73738 (13)	0.23351 (10)	0.58104 (7)	0.0205 (2)	
H11a	0.7246 (10)	0.2324 (3)	0.6387 (2)	0.0308 (4)*	
H11b	0.6775 (7)	0.17259 (13)	0.5473 (3)	0.0308 (4)*	
H11c	0.8397 (3)	0.2197 (4)	0.5830 (5)	0.0308 (4)*	
C2	0.17288 (12)	0.08417 (9)	0.42860 (7)	0.0147 (2)	
C1	0.21122 (12)	0.08264 (9)	0.34140 (7)	0.0149 (2)	
B5	0.33450 (13)	0.14839 (11)	0.42313 (8)	0.0167 (2)	
H5	0.44690 (13)	0.11671 (11)	0.45362 (8)	0.0201 (3)*	
B6	0.22452 (13)	0.21212 (11)	0.48302 (8)	0.0158 (2)	
H6	0.26690 (13)	0.22484 (11)	0.55298 (8)	0.0190 (3)*	
B1	0.04205 (13)	0.16869 (11)	0.43443 (8)	0.0147 (2)	
B2	−0.01661 (13)	0.23783 (11)	0.33083 (8)	0.0150 (2)	
B3	0.10220 (13)	0.16468 (11)	0.27283 (8)	0.0153 (2)	
B4	0.28625 (13)	0.20985 (11)	0.32130 (8)	0.0161 (2)	
H4a	0.36949 (13)	0.22198 (11)	0.28446 (8)	0.0193 (3)*	
B9	0.28663 (13)	0.29761 (11)	0.40908 (8)	0.0161 (2)	
H9	0.36735 (13)	0.36940 (11)	0.42941 (8)	0.0193 (3)*	
B7	0.10328 (13)	0.30870 (10)	0.41803 (8)	0.0147 (2)	
H7a	0.06430 (13)	0.38730 (10)	0.44608 (8)	0.0176 (3)*	
B8	0.14080 (13)	0.30817 (11)	0.31390 (8)	0.0154 (2)	
H8	0.12680 (13)	0.38547 (11)	0.27096 (8)	0.0185 (3)*	
H2	0.1878 (15)	0.0099 (13)	0.4581 (9)	0.019 (3)*	
H2a	−0.1222 (16)	0.2751 (13)	0.2999 (9)	0.025 (4)*	
H1	0.2458 (16)	0.0102 (14)	0.3259 (9)	0.024 (4)*	
H1a	−0.0255 (15)	0.1466 (12)	0.4776 (9)	0.020 (3)*	
H3	0.0733 (16)	0.1437 (13)	0.2064 (9)	0.022 (4)*	
H2b	−0.0116 (18)	0.1414 (15)	0.3104 (10)	0.038 (4)*	

### Atomic displacement parameters (Å^2^)

**Table:**

	*U*^11^	*U*^22^	*U*^33^	*U*^12^	*U*^13^	*U*^23^
N1	0.0161 (4)	0.0128 (4)	0.0155 (4)	0.0003 (3)	0.0040 (3)	0.0013 (3)
N2	0.0157 (4)	0.0125 (4)	0.0168 (5)	0.0006 (3)	0.0049 (3)	−0.0002 (3)
C3	0.0226 (6)	0.0218 (6)	0.0174 (5)	−0.0036 (4)	0.0080 (4)	0.0018 (4)
C6	0.0191 (5)	0.0163 (5)	0.0208 (6)	−0.0031 (4)	0.0010 (4)	−0.0010 (4)
C5	0.0166 (5)	0.0112 (5)	0.0158 (5)	0.0031 (4)	0.0045 (4)	0.0019 (4)
C4	0.0164 (5)	0.0134 (5)	0.0191 (5)	0.0000 (4)	0.0013 (4)	−0.0006 (4)
C7	0.0149 (5)	0.0131 (5)	0.0208 (5)	−0.0009 (4)	0.0025 (4)	−0.0001 (4)
C8	0.0201 (5)	0.0158 (5)	0.0161 (5)	0.0014 (4)	0.0091 (4)	−0.0003 (4)
C9	0.0205 (5)	0.0179 (5)	0.0168 (5)	0.0059 (4)	0.0081 (4)	0.0017 (4)
C10	0.0174 (5)	0.0179 (5)	0.0142 (5)	0.0033 (4)	0.0045 (4)	−0.0011 (4)
C11	0.0258 (6)	0.0212 (6)	0.0150 (5)	0.0065 (4)	0.0059 (4)	0.0013 (4)
C2	0.0176 (5)	0.0120 (5)	0.0151 (5)	0.0005 (4)	0.0055 (4)	0.0016 (4)
C1	0.0166 (5)	0.0129 (5)	0.0165 (5)	0.0003 (4)	0.0067 (4)	−0.0014 (4)
B5	0.0138 (5)	0.0173 (6)	0.0187 (6)	0.0015 (4)	0.0035 (4)	0.0006 (5)
B6	0.0175 (6)	0.0153 (6)	0.0142 (5)	0.0010 (4)	0.0032 (4)	−0.0003 (4)
B1	0.0152 (5)	0.0145 (6)	0.0156 (6)	0.0002 (4)	0.0060 (4)	0.0013 (4)
B2	0.0128 (5)	0.0152 (6)	0.0167 (6)	0.0004 (4)	0.0033 (4)	0.0007 (4)
B3	0.0159 (6)	0.0161 (6)	0.0146 (5)	−0.0017 (4)	0.0052 (4)	−0.0005 (4)
B4	0.0150 (5)	0.0167 (6)	0.0180 (6)	−0.0015 (4)	0.0067 (4)	0.0000 (5)
B9	0.0144 (5)	0.0156 (6)	0.0178 (6)	−0.0021 (4)	0.0034 (4)	−0.0005 (4)
B7	0.0161 (6)	0.0126 (5)	0.0157 (5)	0.0006 (4)	0.0048 (4)	−0.0007 (4)
B8	0.0169 (6)	0.0139 (5)	0.0157 (6)	−0.0011 (4)	0.0047 (4)	0.0018 (4)

### Geometric parameters (Å, º)

**Table:**

N1—C3	1.4608 (14)	C1—B5	1.7125 (16)
N1—C5	1.3382 (14)	C1—B3	1.6219 (16)
N1—C4	1.3819 (14)	C1—B4	1.6986 (16)
N2—C5	1.3367 (14)	C1—H1	0.955 (16)
N2—C7	1.3836 (14)	B5—H5	1.1200
N2—C8	1.4798 (13)	B5—B6	1.7656 (17)
C3—H3a	0.9800	B5—B4	1.7574 (18)
C3—H3b	0.9800	B5—B9	1.7778 (18)
C3—H3c	0.9800	B6—H6	1.1200
C6—H6a	0.9800	B6—B1	1.7873 (17)
C6—H6b	0.9800	B6—B9	1.7732 (18)
C6—H6c	0.9800	B6—B7	1.7524 (17)
C6—C5	1.4827 (15)	B1—B2	1.8238 (17)
C4—H4	0.9500	B1—B7	1.7584 (17)
C4—C7	1.3451 (16)	B1—H1a	1.099 (14)
C7—H7	0.9500	B2—B3	1.8538 (17)
C8—H8a	0.9900	B2—B7	1.7796 (17)
C8—H8b	0.9900	B2—B8	1.7868 (17)
C8—C9	1.5208 (15)	B2—H2a	1.091 (15)
C9—H9a	0.9900	B2—H2b	1.165 (17)
C9—H9b	0.9900	B3—B4	1.8056 (17)
C9—C10	1.5246 (14)	B3—B8	1.7862 (17)
C10—H10a	0.9900	B3—H3	1.077 (14)
C10—H10b	0.9900	B3—H2b	1.401 (16)
C10—C11	1.5236 (15)	B4—H4a	1.1200
C11—H11a	0.9800	B4—B9	1.7536 (18)
C11—H11b	0.9800	B4—B8	1.7693 (18)
C11—H11c	0.9800	B9—H9	1.1200
C2—C1	1.5585 (14)	B9—B7	1.7938 (17)
C2—B5	1.7305 (16)	B9—B8	1.8046 (18)
C2—B6	1.7263 (16)	B7—H7a	1.1200
C2—B1	1.6030 (16)	B7—B8	1.8254 (17)
C2—H2	0.975 (15)	B8—H8	1.1200
			
C5—N1—C3	126.02 (10)	B6—B1—C2	60.93 (7)
C4—N1—C3	124.66 (10)	B2—B1—C2	105.57 (8)
C4—N1—C5	109.30 (9)	B2—B1—B6	108.80 (8)
C7—N2—C5	108.90 (9)	B7—B1—C2	104.76 (8)
C8—N2—C5	127.11 (9)	B7—B1—B6	59.23 (7)
C8—N2—C7	123.89 (9)	B7—B1—B2	59.54 (7)
H3a—C3—N1	109.5	H1a—B1—C2	119.3 (7)
H3b—C3—N1	109.5	H1a—B1—B6	116.2 (7)
H3b—C3—H3a	109.5	H1a—B1—B2	127.3 (7)
H3c—C3—N1	109.5	H1a—B1—B7	125.5 (7)
H3c—C3—H3a	109.5	B3—B2—B1	101.16 (8)
H3c—C3—H3b	109.5	B7—B2—B1	58.40 (7)
H6b—C6—H6a	109.5	B7—B2—B3	105.48 (8)
H6c—C6—H6a	109.5	B8—B2—B1	105.76 (8)
H6c—C6—H6b	109.5	B8—B2—B3	58.73 (7)
C5—C6—H6a	109.5	B8—B2—B7	61.57 (7)
C5—C6—H6b	109.5	H2a—B2—B1	128.9 (8)
C5—C6—H6c	109.5	H2a—B2—B3	123.6 (8)
N2—C5—N1	107.59 (9)	H2a—B2—B7	121.1 (8)
C6—C5—N1	124.98 (10)	H2a—B2—B8	117.4 (8)
C6—C5—N2	127.42 (10)	H2b—B2—B1	79.9 (8)
H4—C4—N1	126.59 (6)	H2b—B2—B3	49.1 (8)
C7—C4—N1	106.82 (10)	H2b—B2—B7	127.0 (8)
C7—C4—H4	126.59 (7)	H2b—B2—B8	106.9 (8)
C4—C7—N2	107.39 (9)	H2b—B2—H2a	110.1 (11)
H7—C7—N2	126.31 (6)	B2—B3—C1	106.17 (8)
H7—C7—C4	126.31 (7)	B4—B3—C1	59.13 (7)
H8a—C8—N2	109.56 (6)	B4—B3—B2	107.22 (8)
H8b—C8—N2	109.56 (6)	B8—B3—C1	104.23 (8)
H8b—C8—H8a	108.1	B8—B3—B2	58.76 (7)
C9—C8—N2	110.48 (9)	B8—B3—B4	59.02 (7)
C9—C8—H8a	109.56 (6)	H3—B3—C1	121.3 (8)
C9—C8—H8b	109.56 (6)	H3—B3—B2	125.8 (8)
H9a—C9—C8	108.98 (6)	H3—B3—B4	118.4 (8)
H9b—C9—C8	108.98 (6)	H3—B3—B8	124.1 (8)
H9b—C9—H9a	107.8	H2b—B3—C1	90.9 (7)
C10—C9—C8	113.02 (9)	H2b—B3—B2	38.9 (7)
C10—C9—H9a	108.98 (6)	H2b—B3—B4	129.5 (7)
C10—C9—H9b	108.98 (6)	H2b—B3—B8	96.9 (7)
H10a—C10—C9	109.39 (6)	H2b—B3—H3	111.8 (10)
H10b—C10—C9	109.39 (6)	B5—B4—C1	59.38 (7)
H10b—C10—H10a	108.0	B3—B4—C1	55.04 (6)
C11—C10—C9	111.23 (9)	B3—B4—B5	106.77 (8)
C11—C10—H10a	109.39 (6)	H4a—B4—C1	127.03 (5)
C11—C10—H10b	109.39 (6)	H4a—B4—B5	120.62 (6)
H11a—C11—C10	109.5	H4a—B4—B3	122.79 (5)
H11b—C11—C10	109.5	B9—B4—C1	104.60 (8)
H11b—C11—H11a	109.5	B9—B4—B5	60.84 (7)
H11c—C11—C10	109.5	B9—B4—B3	108.87 (8)
H11c—C11—H11a	109.5	B9—B4—H4a	120.92 (6)
H11c—C11—H11b	109.5	B8—B4—C1	101.80 (8)
B5—C2—C1	62.50 (7)	B8—B4—B5	109.46 (9)
B6—C2—C1	112.20 (8)	B8—B4—B3	59.94 (7)
B6—C2—B5	61.43 (7)	B8—B4—H4a	122.20 (6)
B1—C2—C1	115.37 (9)	B8—B4—B9	61.62 (7)
B1—C2—B5	117.30 (9)	B6—B9—B5	59.63 (7)
B1—C2—B6	64.82 (7)	B4—B9—B5	59.68 (7)
H2—C2—C1	113.8 (8)	B4—B9—B6	107.80 (9)
H2—C2—B5	112.5 (8)	H9—B9—B5	122.78 (5)
H2—C2—B6	120.6 (8)	H9—B9—B6	122.09 (6)
H2—C2—B1	121.4 (8)	H9—B9—B4	121.72 (6)
B5—C1—C2	63.68 (7)	B7—B9—B5	106.33 (8)
B3—C1—C2	111.49 (8)	B7—B9—B6	58.85 (7)
B3—C1—B5	118.09 (9)	B7—B9—B4	108.02 (8)
B4—C1—C2	112.05 (8)	B7—B9—H9	122.14 (6)
B4—C1—B5	62.02 (7)	B8—B9—B5	106.97 (8)
B4—C1—B3	65.83 (7)	B8—B9—B6	107.88 (8)
H1—C1—C2	115.4 (9)	B8—B9—B4	59.62 (7)
H1—C1—B5	112.3 (9)	B8—B9—H9	121.63 (5)
H1—C1—B3	122.0 (9)	B8—B9—B7	60.97 (7)
H1—C1—B4	120.8 (9)	B1—B7—B6	61.21 (7)
C1—B5—C2	53.82 (6)	B2—B7—B6	112.47 (8)
H5—B5—C2	126.83 (5)	B2—B7—B1	62.06 (7)
H5—B5—C1	126.11 (5)	B9—B7—B6	59.99 (7)
B6—B5—C2	59.17 (7)	B9—B7—B1	108.35 (8)
B6—B5—C1	103.31 (8)	B9—B7—B2	109.93 (8)
B6—B5—H5	121.61 (6)	H7a—B7—B6	120.08 (6)
B4—B5—C2	101.56 (8)	H7a—B7—B1	121.43 (5)
B4—B5—C1	58.60 (7)	H7a—B7—B2	119.10 (5)
B4—B5—H5	122.74 (6)	H7a—B7—B9	121.49 (6)
B4—B5—B6	107.97 (8)	B8—B7—B6	107.87 (8)
B9—B5—C2	102.58 (8)	B8—B7—B1	106.89 (8)
B9—B5—C1	102.99 (8)	B8—B7—B2	59.41 (7)
B9—B5—H5	123.83 (5)	B8—B7—B9	59.81 (7)
B9—B5—B6	60.05 (7)	B8—B7—H7a	123.25 (5)
B9—B5—B4	59.47 (7)	B3—B8—B2	62.51 (7)
B5—B6—C2	59.40 (7)	B4—B8—B2	111.87 (8)
H6—B6—C2	128.17 (5)	B4—B8—B3	61.04 (7)
H6—B6—B5	120.64 (6)	B9—B8—B2	109.11 (8)
B1—B6—C2	54.26 (6)	B9—B8—B3	107.48 (8)
B1—B6—B5	106.55 (8)	B9—B8—B4	58.76 (7)
B1—B6—H6	123.06 (5)	B7—B8—B2	59.02 (7)
B9—B6—C2	102.94 (8)	B7—B8—B3	106.41 (8)
B9—B6—B5	60.31 (7)	B7—B8—B4	105.96 (8)
B9—B6—H6	121.77 (6)	B7—B8—B9	59.23 (7)
B9—B6—B1	107.98 (8)	H8—B8—B2	119.15 (5)
B7—B6—C2	99.97 (8)	H8—B8—B3	121.34 (5)
B7—B6—B5	108.69 (8)	H8—B8—B4	121.15 (6)
B7—B6—H6	123.01 (6)	H8—B8—B9	122.36 (5)
B7—B6—B1	59.56 (7)	H8—B8—B7	124.10 (5)
B7—B6—B9	61.16 (7)	B3—H2b—B2	92.0 (11)
