# Supplementary figures and images for: Crystal structure of 1-butyl-2,3-di­methyl­imidazolium dicarba-7,8-nido-undeca­borate
Source: Acta Crystallogr E Crystallogr Commun. 2015 Feb 13;71(Pt 3):o183. doi: 10.1107/S2056989015002765 (PMC4350724; doi:10.1107/S2056989015002765)

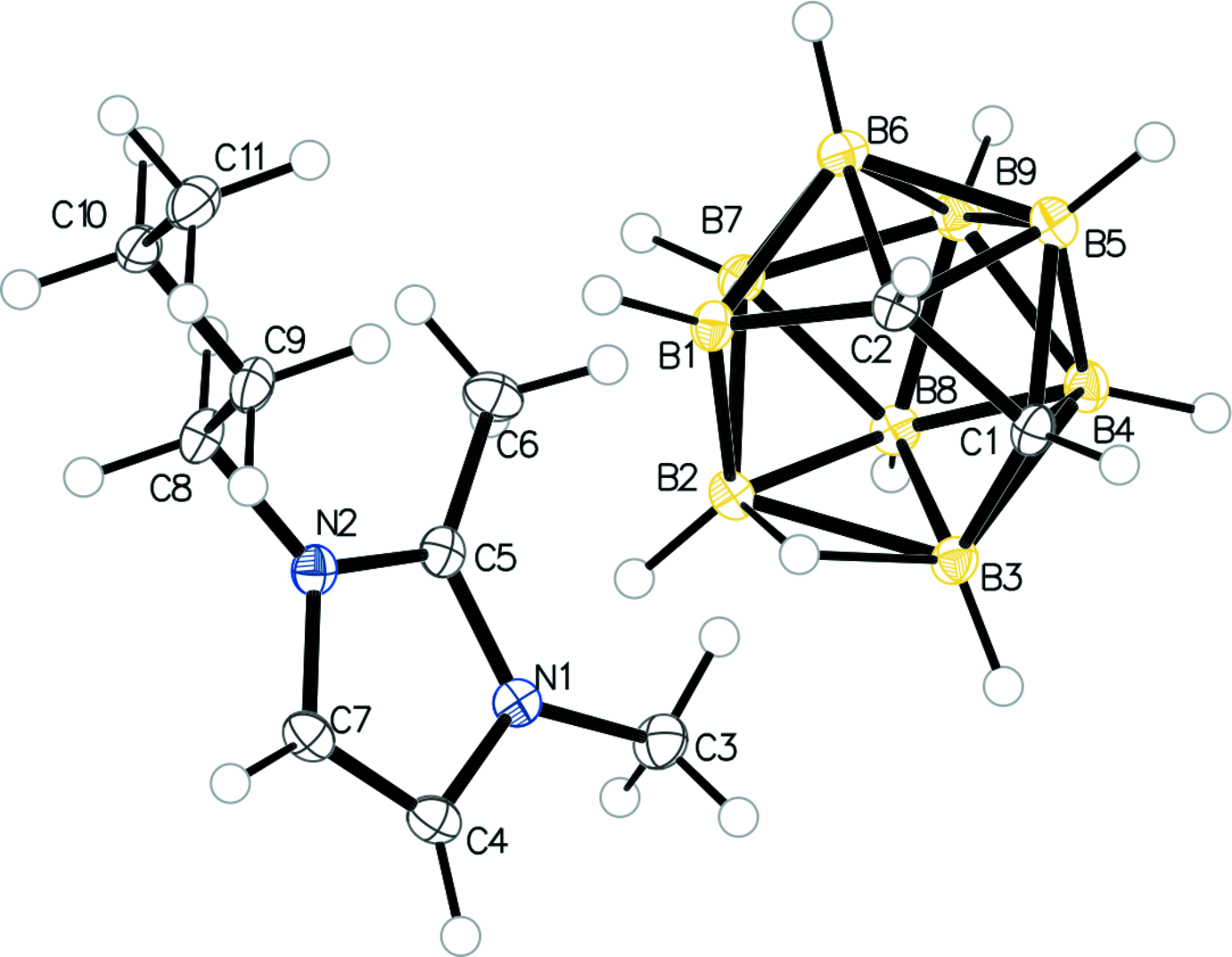

Supplement: Supplementary file 3 [file e-71-0o183-fig1.tif]

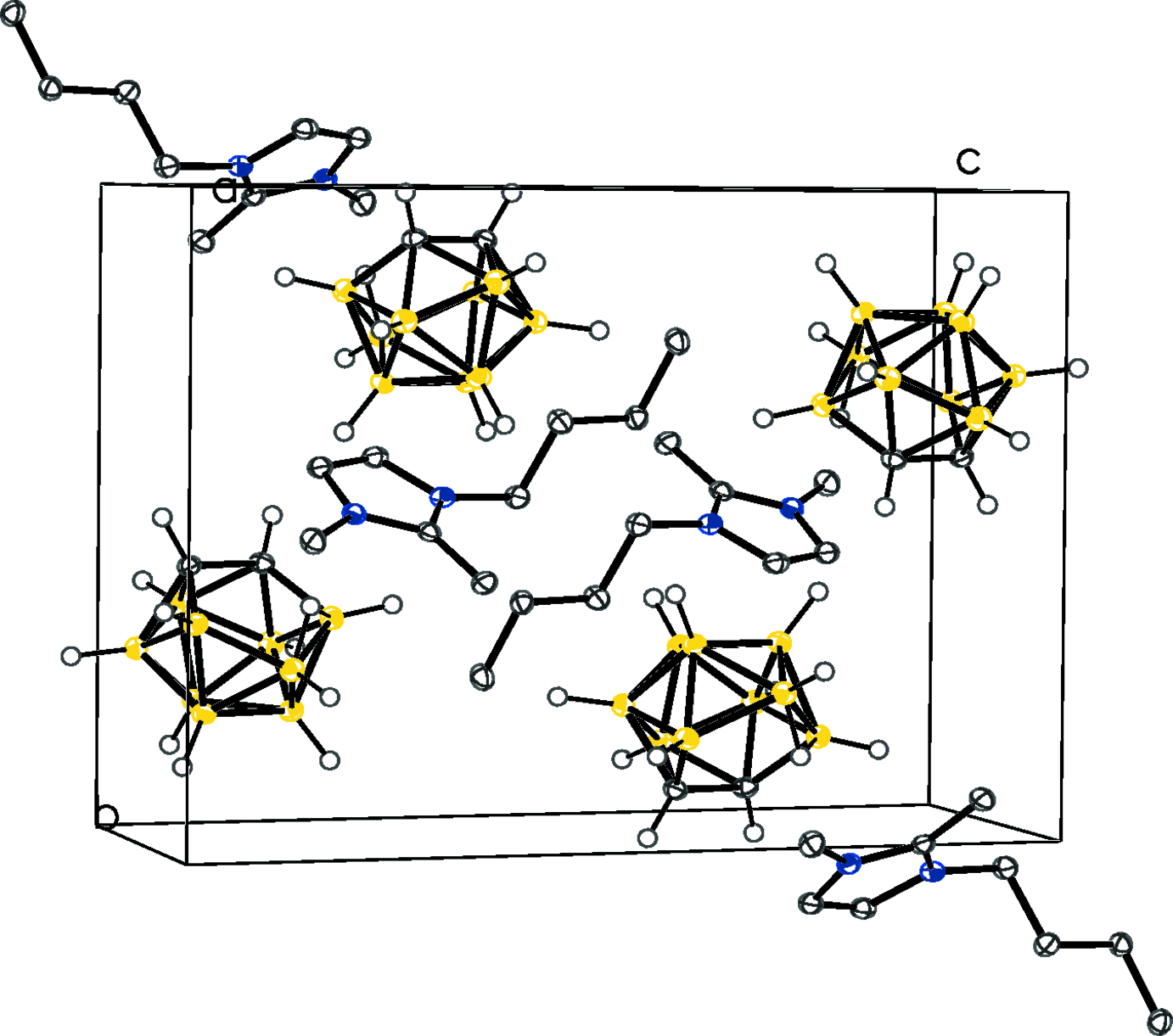

Supplement: Supplementary file 4 [file e-71-0o183-fig2.tif]

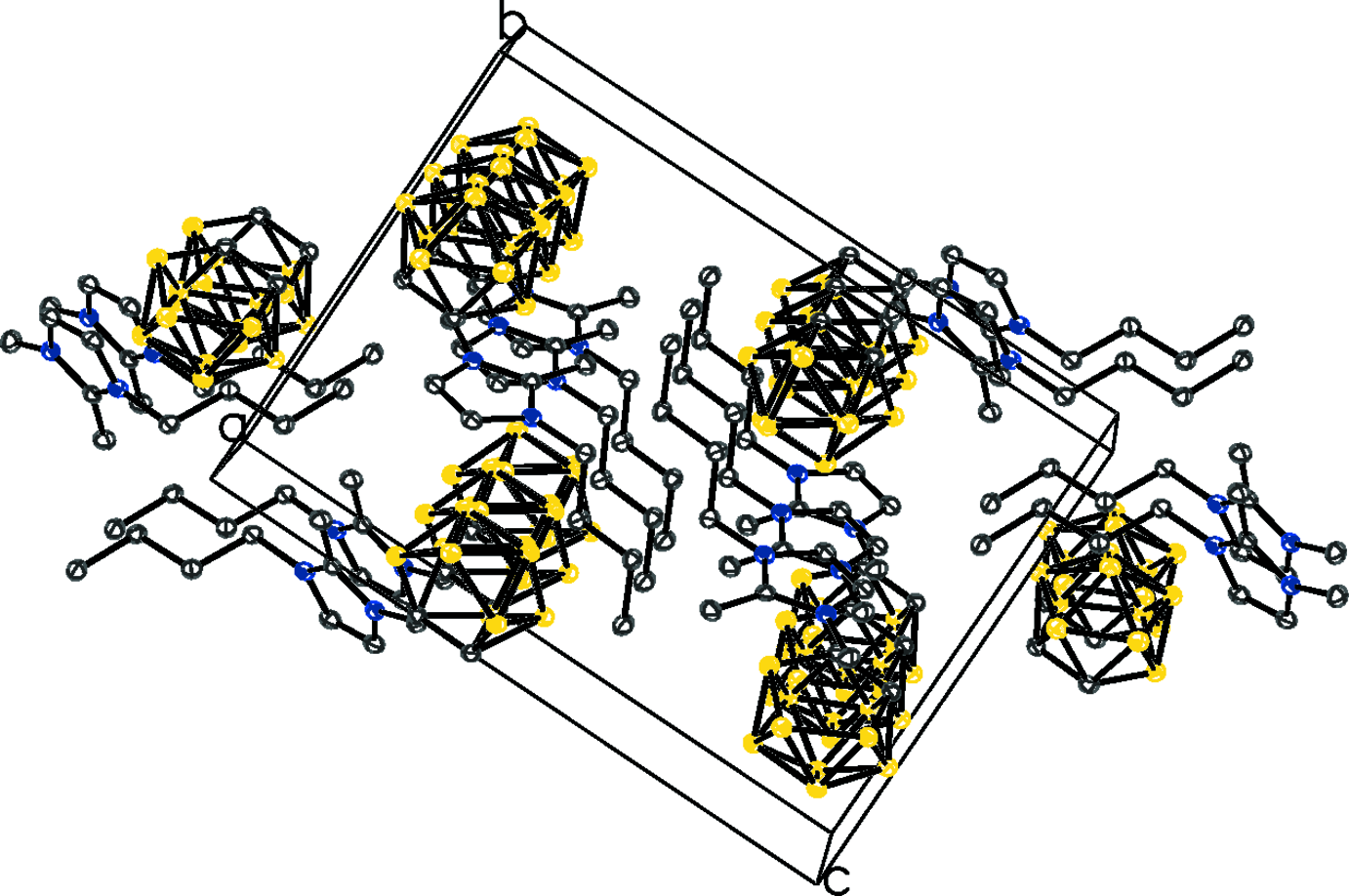

Supplement: Supplementary file 5 [file e-71-0o183-fig3.tif]
